# Broad spectrum vasopressors: a new approach to the initial management of septic shock?

**DOI:** 10.1186/s13054-019-2420-y

**Published:** 2019-04-16

**Authors:** Lakhmir S. Chawla, Marlies Ostermann, Lui Forni, George F. Tidmarsh

**Affiliations:** 1 Veterans Affairs Medical Center, 3350 La Jolla Village Dr, San Diego, CA 92161 USA; 2 La Jolla Pharmaceutical Company, San Diego, CA USA; 3 King’s College London, Guy’s & St Thomas’ Hospital, London, UK; 4 Department of Clinical and Experimental Medicine, Faculty of Health Sciences, University of Surrey, Guildford, UK; 5 Intensive Care Unit, Royal Surrey County Hospital NHS Foundation Trust, Guildford, UK; 6 Stanford University School of Medicine, Palo Alto, CA 94305 USA

**Keywords:** Septic shock, Catecholamines, Vasopressin, Angiotensin II, Sensitivity

## Abstract

The mainstay of hemodynamic treatment of septic shock is fluid resuscitation followed by vasopressors where fluids alone are insufficient to achieve target blood pressure. Norepinephrine, a catecholamine, is the first-line vasopressor used worldwide but given that all routinely used catecholamines target the same adrenergic receptors, many clinicians may add a non-catecholamine vasopressor where refractory hypotension due to septic shock is present. However, the timing of this additional intervention is variable. This decision is based on three key factors: availability, familiarity, and safety profile. In our opinion, further consideration should be potential vasopressor response because following appropriate volume resuscitation, the response to different vasopressor classes is neither uniform nor predictable. Critically ill patients who are non-responders to high-dose catecholamines have a dismal outcome. Similarly, patients have a variable response to non-catecholamine agents including vasopressin and angiotensin II: but where patients exhibit a blood pressure response the outcomes are improved over non-responders. This variable responsiveness to vasopressors is similar to the clinical approach of anti-microbial sensitivity. In this commentary, the authors propose the concept of “broad spectrum vasopressors” wherein patients with septic shock are started on multiple vasopressors with a different mechanism of action simultaneously while the vasopressor sensitivity is assessed. Once the vasopressor sensitivities are assessed, then the vasopressors are ‘de-escalated’ accordingly. We believe that this concept may offer a new approach to the treatment of septic shock.

## Background

Sepsis remains the most common cause of vasodilatory shock worldwide. International consensus guidelines describe specific recommendations regarding treatment. These include the timing of important interventions comprising blood culture collection, initiation of broad-spectrum antibiotics, blood glucose targets, use of steroids, and restoration of optimal hemodynamic status [1]. The mainstay of treatment with regard to restoring and maintaining optimal hemodynamic status is rapid and appropriate fluid bolus therapy (FBT) which, if insufficient, is followed by vasopressor therapy to maintain an acceptable mean arterial pressure (MAP). Despite this approach being a cornerstone of therapeutic guidelines, there is a lack of high-quality evidence demonstrating a survival benefit associated with the use of one vasopressor over another [2]. Although current consensus guidelines recommend norepinephrine as the first-line vasopressor, both selection and timing of second-line therapy in refractory hypotension due to septic shock is highly variable. Indeed, in a recent survey of practice, only 14% of respondents cited a predefined dose of the first agent as the stimulus for additional therapy [3, 4]. Selection of the vasopressor agent is also variable and further complicated by the recent data related to a “new” vasopressor, angiotensin II, which is currently only available in the USA [5, 6].

## Main text

Given that all routinely used catecholamines target the same adrenergic receptors, most clinicians are inclined to add a non-catecholamine vasopressor to treat patients with refractory hypotension due to septic shock, a decision based on three key factors: availability (a relatively rigid constraint), familiarity (often governed by previous practice), and safety profile. In our opinion, further consideration should be potential vasopressor response. Following appropriate volume resuscitation, the response to different vasopressor classes is neither uniform nor predictable. Furthermore, the responsiveness to a vasopressor may impact the outcome. Indeed, norepinephrine, recognized as the first-line vasopressor, often demonstrates a variable response which may be due to various factors which include pre-existing therapy/medications, genetics, the underlying pathophysiology of septic shock, and/or receptor responsiveness [7–10]. Both human and pre-clinical data demonstrate that septic shock impairs sympathetic modulation of the heart and vasculature [7]. In fact, septic shock patients who maintain adrenergic responsiveness have better outcomes [9]. Similarly, non-catecholamine vasopressors including vasopressin and angiotensin II can be affected by concomitant medications, genetics, and altered receptor responsiveness as a consequence of inflammation and sepsis [10–12]. The net effects of all of these parameters are difficult to compute at the bedside, but the key issue is whether responsiveness to a vasopressor impacts outcomes.

**Fig. 1:**
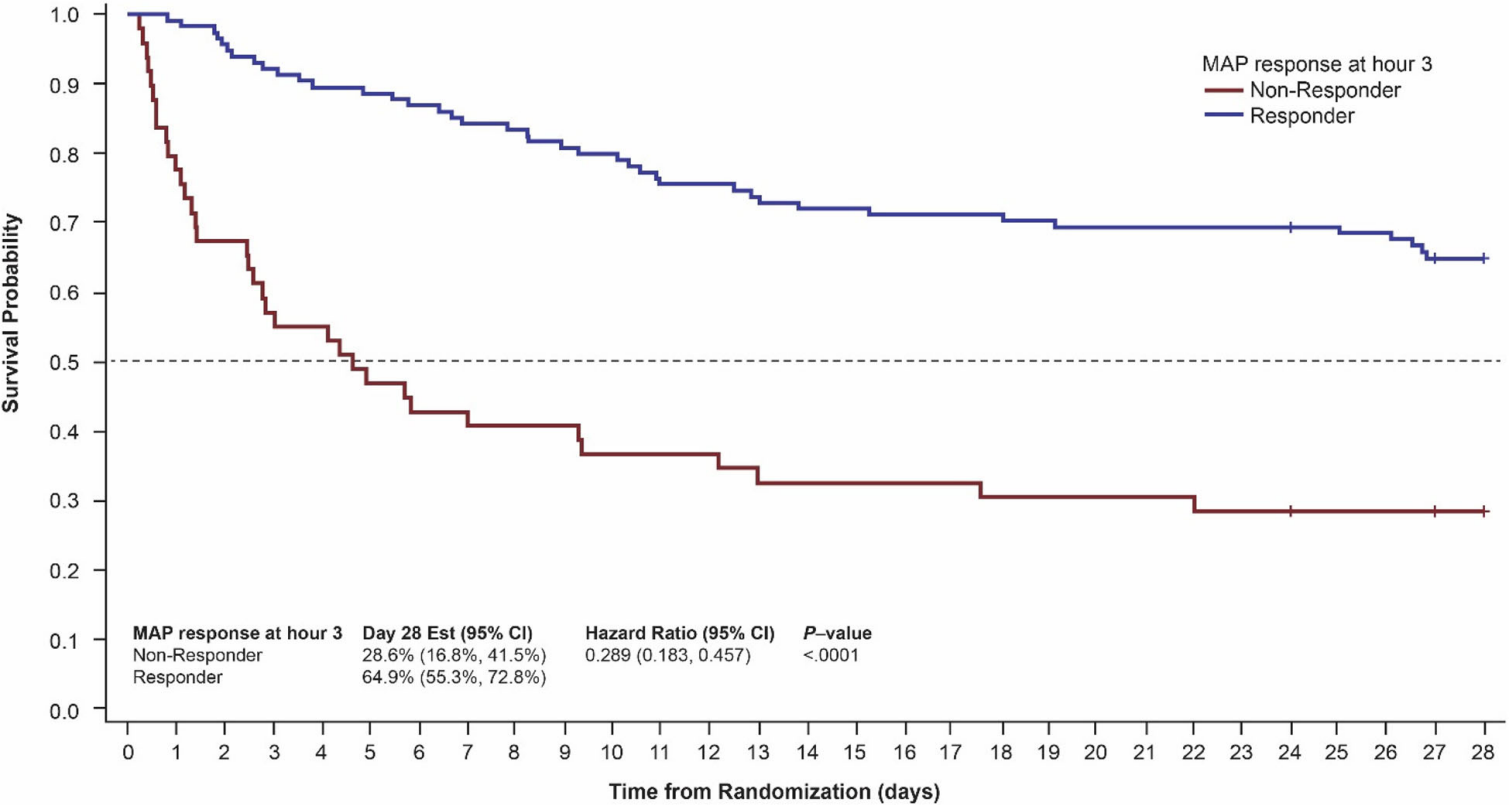
Survival probability by MAP response at hour 3 for patients in the ATHOS-3 trial

Non-responders to high-dose catecholamines have a dismal outcome [13]. In terms of non-catecholamine agents, less than 50% of patients demonstrate a MAP response to low-dose vasopressin with this group having a significantly better survival than those that fail to respond [14] (Table 1). Similarly, approximately 70% of patients who receive angiotensin II have a MAP response. [6, 15] In a responder analysis, the ATHOS-3 study showed that a responder’s chance of survival is significantly better than that of patients who fail to respond to angiotensin II [6, 15] (Table 1, Fig. 1). It follows that in patients with septic shock, the choice of vasopressor should be governed by the patient’s likelihood of responding and the sensitivity to treatment. This notion is in keeping with current antimicrobial therapy paradigm wherein clinicians obtain cultures and start broad-spectrum antibiotics with the intention of de-escalating the antibiotics once the causative organism is identified.

**Table 1:** Outcomes Assessed by MAP Response to Vasopressin or Angiotensin II

	Vasopressin [14]			Angiotensin II [6]		
	Responder 45% (*n* = 426)	Non-responder 55% (*n* = 512)	*P* value	Responder 69.9% (*n* = 114)	Non-responder 30.1% (*n* = 49)	*P* value
In-patient mortality (%)	56.6%	71.7%	< 0.001	35%	71%	< 0.0001
Catecholamine dose (mcg/kg/min)	0.35 ± 0.25	0.33 ± 0.27	0.18	0.32 ± 0.32	0.53 ± 0.39	0.0002
MAP (mmHg)	69 ± 12	65 ± 12	< 0.001	67.1 ± 5.21	64.8 ± 5.02	0.0087
Steroids (%)	58.9	62.5	0.26	55.3	57.1	0.8647
Lactate (mmol/L)	4.0 ± 3.6	5.4 ± 4.8	< 0.001	3.43 ± 2.43	5.80 ± 6.01	0.013
SOFA score	13 ± 4	12 ± 3	0.49	11.65 ± 2.78	12.06 ± 2.99	0.4313
Age, years	62 ± 14	61 ± 15	0.17	61.7 ± 15.32	63.1 ± 16.33	0.4278

## Conclusion

We propose the notion of “broad spectrum vasopressors” wherein patients with septic shock are started on multiple vasopressors with a different mechanism of action simultaneously while the vasopressor sensitivity is assessed. Vasopressor sensitivity could be assessed by sequential removal of vasopressors or developing a vasopressor sensitivity panel. Once the vasopressor sensitivities are assessed, then the vasopressors are de-escalated accordingly. However, this concept is hampered by several issues. Firstly, there is currently no bedside test that predicts the blood pressure response to catecholamines, vasopressin, or angiotensin II. Secondly, not all of these vasopressors are currently available worldwide due to either a lack of regulatory approval or cost considerations. Thirdly, there are no prospective data supporting this approach. Despite these hurdles, we feel that this is a testable hypothesis: Does time to sensitive vasopressor response improve outcomes in septic shock? We suggest this is a question worth answering and may prove an essential approach in managing these critically ill individuals.

## Abbreviations

FBT: Fluid bolus therapy
MAP: Mean arterial pressure
